# Cam Design and Pin Defect Detection of Cam Pin Insertion Machine in IGBT Packaging

**DOI:** 10.3390/mi16070829

**Published:** 2025-07-20

**Authors:** Wenchao Tian, Pengchao Zhang, Mingfang Tian, Si Chen, Haoyue Ji, Bingxu Ma

**Affiliations:** 1 State Key Laboratory of Electromechanical Integrated Manufacturing of High-Performance Electronic Equipment, Xidian University, Xi’an 710071, China; 2 School of Mechano-Electronic Engineering, Xidian University, Xi’an 710071, China; 3 Wuxi China Resources Huajing Microelectronics Co., Ltd., Wuxi 214061, China; 4 The Fifth Electronics Research Institute of Ministry of Industry and Information Technology, Guangzhou 510000, China; 5 The 58th Research Institute of China Electronics Technology Group Corporation, Wuxi 214062, China; 6 The Science and Technology on Reliability Physics and Application of Electronic Component Laboratory, China Electronic Product Reliability and Environmental Testing Research Institute, Guangzhou 510000, China

**Keywords:** IGBT, pin insert machine, cam pin mechanism, seventh degree polynomial, machine vision, defect detection

## Abstract

Packaging equipment plays a crucial role in the semiconductor industry by enhancing product quality and reducing labor costs through automation. Research was conducted on IGBT module packaging equipment (an automatic pin insertion machine) during the pin assembly process of insulated gate bipolar transistor (IGBT) modules to improve productivity and product quality. First, the manual pin assembly process was divided into four stages: feeding, stabilizing, clamping, and inserting. Each stage was completed by separate cams, and corresponding step timing diagrams are drawn. The profiles of the four cams were designed and verified through theoretical calculations and kinematic simulations using a seventh-degree polynomial curve fitting method. Then, image algorithms were developed to detect pin tilt defects, pin tip defects, and to provide visual guidance for pin insertion. Finally, a pin insertion machine and its human–machine interaction interface were constructed. On-machine results show that the pin cutting pass rate reached 97%, the average insertion time for one pin was 2.84 s, the pass rate for pin insertion reached 99.75%, and the pin image guidance accuracy was 0.02 mm. Therefore, the designed pin assembly machine can reliably and consistently perform the pin insertion task, providing theoretical and experimental insights for the automated production of IGBT modules.

## 1. Introduction

**Figure 1:**
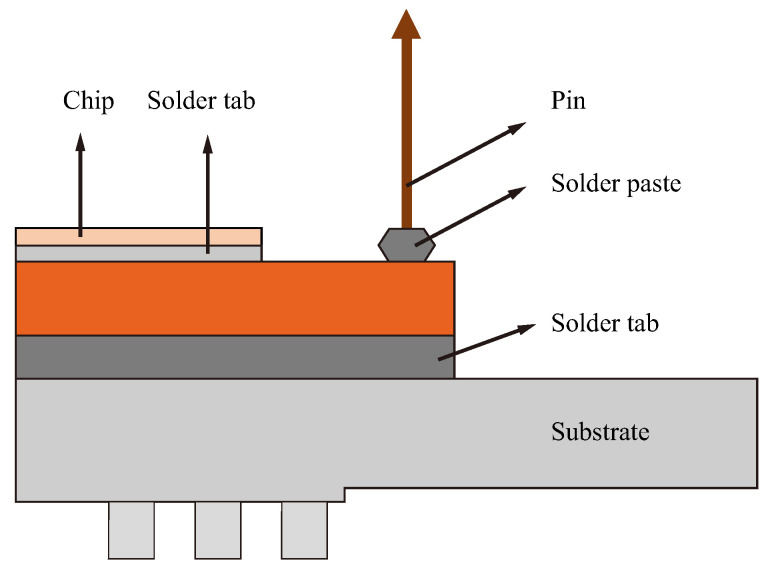
IGBT module pin cutaway schematic diagram.

Insulated gate bipolar transistor (IGBT) modules are characterized by their fast switching speeds and low losses. They are widely utilized in rail transportation, new energy vehicles, smart grids, and daily electrical appliances [1,2,3]. The cross-sectional view of the IGBT package is shown in Figure 1. However, IGBTs are susceptible to material structural deformation, inadequate pin contact, electrical open circuits, short circuits, and other faults. These issues can result in thermal failure, electrical failure, vibration-induced failure, and fatigue failure, ultimately leading to packaging failure [4]. Pins of IGBT serve as a bridge between the IGBT and external electronic devices. Packaging problems for pins include defects such as solder voids, cracks, desoldering, stains, and missing pins [5,6]. In the packaging of IGBT module pins, the automatic high-speed pin insertion machine completes the pin insertion process through the high-speed reciprocating motion of the pins, improving product quality and reducing labor costs [7]. At the same time, approximately 70% of the work of inserting electronic devices into components is performed manually, which is inefficient, costly, and subject to insertion errors [8,9]. Therefore, it is necessary to conduct research on high-speed pin insertion machines to improve the efficiency of IGBT production and enhance fault detection capabilities, thereby increasing product reliability.

Researchers have studied the failure detection of IGBT from various aspects. Lin and Wei obtained the chip junction temperature by precisely measuring the rise time of the IGBT voltage, offering a new method for assessing the health status of IGBT [10]. Cheng proposed a bond wire lift-OFF fault detection method based on the mapping correlation between signal, impedance, and faults to quantitatively evaluate the degradation trend of bond wires [11]. Geng evaluated the condition of the bonding wire and its variation law of IGBT by extracting the switching stress wave (SSW) released by IGBT [12]. Shen proposed an IGBT bonding wire monitoring method that combines feature extraction and condition classification [13]. The aforementioned IGBT failure detection method requires conducting power-on tests on IGBTs, making it challenging to implement directly in the production of IGBTs. In 2017, Lu proposed a method utilizing machine vision technology for pin recognition and offset detection in aviation electrical connectors [14]. The developed system achieves a measurement accuracy of 0.06 mm. In 2023, Zhao developed a connector pin skew detection system utilizing machine vision, achieving a theoretical accuracy of 0.05 mm [15]. In 2023, Chris utilized computer vision and machine learning for real-time automatic detection of socket defects, achieving a validation accuracy of 99.6% [16]. In 2024, Wang designed a pin offset detection algorithm for aerospace electrical connectors, with an actual detection accuracy of 0.09 mm [17]. Therefore, integrating image recognition algorithms into the pin insertion machine is anticipated to achieve real-time pin defect detection during the packaging process.

In 2016, Polverini et al. applied a dual-arm robot to solve the insertion problem. The response characteristics of the robotic system were altered so that it exhibited the desired supple trajectory when in contact with the environment, without using any force sensors [18]. In 2018, Hsien-I Lin used a six-degree-of-freedom robotic arm combining force control, vision servoing, and machine learning to generate a motion trajectory that reduces vibration and shock during high-speed insertion by optimizing the robotic arm’s insertion motion [19]. Robotic arms with four degrees of freedom exhibit adequate point-to-point control for applications where the working range is limited and complex 3D manipulation is not required. The design of motion laws for a higher degree of freedom in robotic arms requires a combination of kinematics and dynamics, considering factors such as singularity analysis and forces [20]. Research on innovative and high-performance products, such as multi-degree-of-freedom robotic arms, contains complex design theories that are difficult to apply quickly in a production environment [21].

Compared with multi-degree-of-freedom robotic arms, the cam mechanism has many advantages such as less space occupation, high speed and reliability, high repeatability, stable transmission timing, and applicability to a variety of motion curves [22]. It is widely used in automated assembly and other fields, such as food, pharmaceutical, beverage packaging machines, etc. To achieve smooth motion at high speeds, cam profiles need to be as smooth as possible in terms of continuous position, continuous velocity, continuous acceleration, continuous jerks, and even higher order smoothness [23,24]. In 2016, Zhou analyzed the displacement function of the follower using the Fourier series to design a cam with less vibration and impact velocity at high speed, but there is a risk of resonance when the number of terms in the Fourier series is too high; therefore, an appropriate number of terms needs to be chosen [25]. In 2019, Nguyen et al. proposed a method for the kinematic design of a cam mechanism followers using non-uniform rational B-splines (NURBSs) which employs NURBS curves to satisfy the kinematic requirements of the follower [24]. Although NURBS curves provide a higher degree of freedom for cam profile curve design, their solution is more complicated and requires considerable computational and optimization work. In 2020, Li proved that the cylindrical cam profile fitted by a fifth-degree polynomial curve has a smaller maximum contact force, which significantly reduces the vibration and shock of the capping machine equipment in high-speed operation [26]. In 2020, Abderazek et al. reported that the target function values of the 3-4-5th movement rules outperform those of the pendulum motion, modified harmonic motion, and the 4-5-6-7th polynomial motion [27]. In 2024, Rao et al. reduced the input torque, cam pitch circle radius, and maximum contact stress required for rotating cams by designing optimization algorithms. Moreover, the objective function value of the 3-4-5th polynomial motion outperforms those of the pendulum motion, modified harmonic motion, and 4-5-6-7th polynomial motion [28]. The polynomial motion law has advantages over other motion laws. With the increase in polynomial order, the continuity of the motion law becomes stronger, satisfying the continuity effects of velocity continuity, acceleration continuity, and even better. In 2023, Güven et al. designed a cylindrical cam mechanism with multiple vertical outputs using computer-aided design software such as SolidWorks and AutoCAD in conjunction with the visual basic for applications (VBA) programming language [29]. Up to now, research on cylindrical and planar cam mechanisms has been relatively comprehensive, including structural form selection, dimensioning, cam profile design, selection of the motion law, curve equation derivation, kinematic characterization, kinematic simulation, force analysis, and fatigue life analysis [30,31]. Moreover, in the fast cam pin insertion machine applicable to the packaging process, the design and analysis of the cam have not yet been fully carried out, which brings vibration, pin jamming, pin waste, and other problems to production.

The purpose of this article is to design an IGBT pin insertion machine and achieve real-time defect detection of pins. First, by analyzing the functions of the pin insertion machine, we determined the timing relationships of each cam within the cam group. The feasibility and applicability of the cam profile were calculated and verified. Next, we employed image recognition algorithms to detect pin tilt, identify pin tip defects, and provide guidance for pin insertion positions. We measured the height of the pins to evaluate the effectiveness of the pin insertion process. Finally, we developed a prototype to validate the design and functionality of the fast pin insertion system. The main contributions are as follows:

1: Decomposition of the process of pin insertion and coordination of the actions of the four cams to achieve pin insertion.

2: Design of visual algorithms to detect pin tip defects and pin tilt defects and provision of visual guidance for pin insertion.

3: Development of a human-computer interaction interface that displays the real-time status of pin defects and visual guidance coordinates while also storing detection results.

## 2. Cam Design in the Pin Insertion Mechanism

On the one hand, the cam design in the pin insertion mechanism should analyze the pin assembly process in the IGBT module assembly production process, and on the other hand, it is necessary to comprehensively consider the coordinated movement between the cams and the timing relationship between the various process actions. The design of the cam pin insertion mechanism mainly includes two parts: process decomposition and cam profile fitting and solving.

### 2.1. Process Decomposition

**Figure 2:**
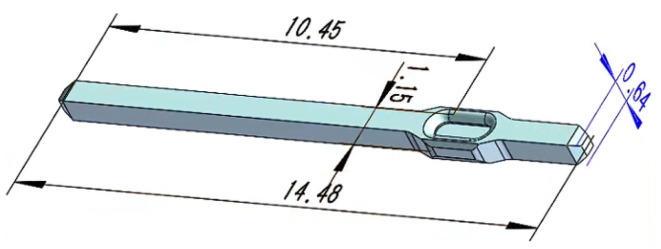
Three-dimensional dimensions of a pin (units: mm).

**Figure 3:**
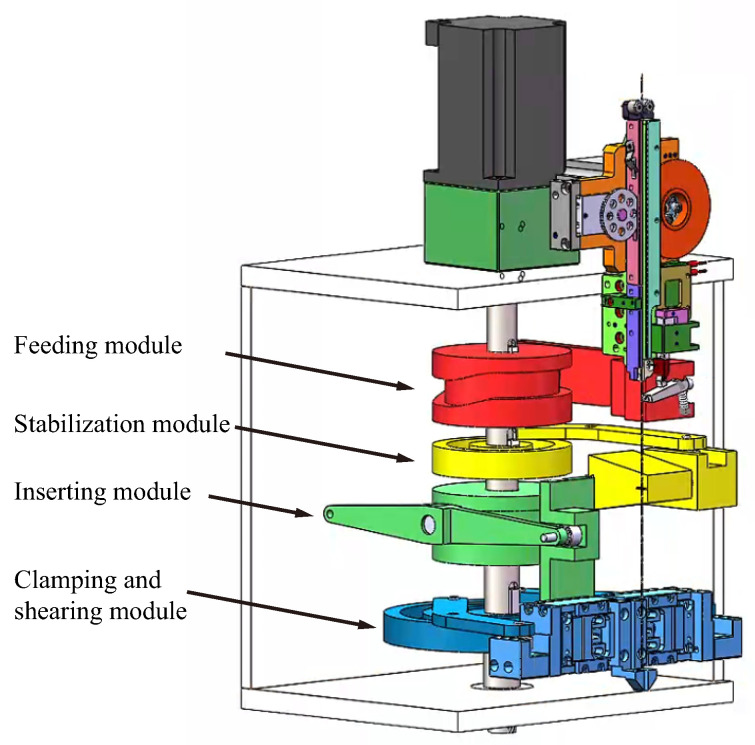
Isometric view of the pin insertion mechanism.

The standard length of the pin of the pin mechanism is 14.48 mm, and the three-dimensional dimensions of the pin are shown in Figure 2. By analyzing the pin assembly behavior in the IGBT module assembly process, the pin assembly process behavior can be divided into four mechanical actions, namely feeding, stabilizing, clamping, and inserting, which correspond to four modules, as shown in Figure 3. The ratio of the 3D model used for theoretical design to the actual object is 1:1. The pin insertion machine completes the feeding, clamping, cutting, and inserting work in coordination with these four modules.

The specific work between the modules is specified below.

**Figure 4:**
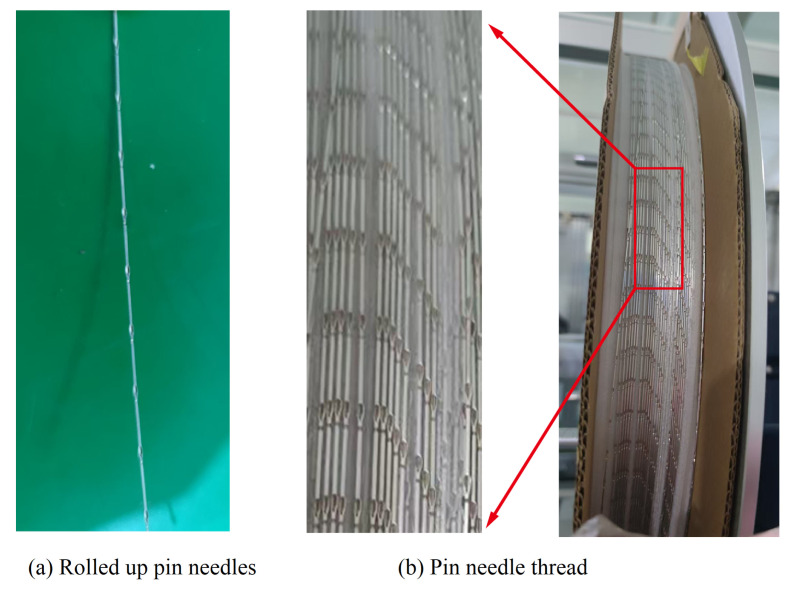
Raw materials for pin.

(1) Feeding module. The pin material is similar to a wire shape, as shown in Figure 4. The feeding mechanism slowly drags the pin material into the feeding track to prepare for the subsequent process.

(2) Stabilization module. The pin feeding speed is controlled to avoid the pin entering the shearing mechanism too fast or too slow. The pause for the clamping mechanism is prepared to shear the pin. When the pin raw material is about to be cut, the material stabilization module suspends the pin raw material.

(3) Clamping and shearing module. When the pin raw material enters the clamping and shearing module, the stabilizing mechanism suspends the feeding of the pin raw material. Then, the clamping mechanism cuts the pin raw material and clamps the pin. The clamping module consists of a left collet and a right cutting knife. The left collet is prepared in advance before the stabilizing module works.

(4) Inserting module. When the clamping module completes pin-cutting work and the pin is clamped, the pin insertion mechanism inserts the pin into the corresponding hole to complete the pin insertion work.

Under the premise of achieving reciprocating short-range displacement work, the pin insertion mechanism also needs to meet various requirements such as stable mechanical structure motion timing, small occupied space, low equipment vibration, fast motion speed, and high output accuracy. The cam mechanism has many advantages, such as occupying less space, high speed and reliability, high repetition accuracy, and stable transmission timing, and it is suitable for various types of motion. Therefore, a cylindrical cam linkage mechanism is adopted to design the feeding module and the pin insertion module. The material stabilization module and clamping module are designed via a planar cam linkage mechanism.

**Figure 5:**
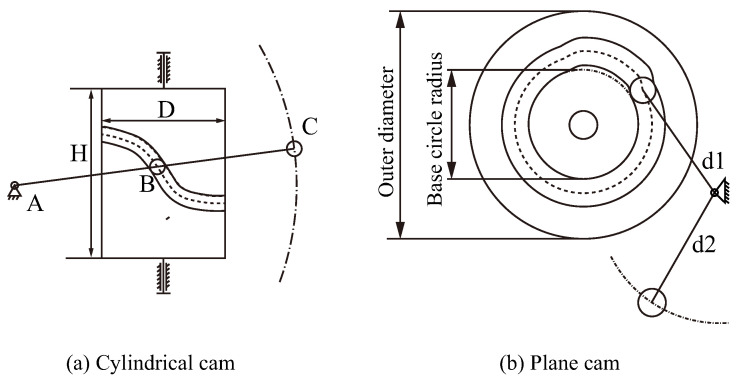
Cylindrical and plane cam pendulum mechanism sketch.

The feeding cam adopts a cylindrical cam pendulum mechanism, as shown in Figure 5a. The roller on the cylindrical cam is located at point B of the pendulum, $L_{AB}=100\;\mathrm{mm}$ and $L_{BC}=120\;\mathrm{mm}$. Then, the pendulum length $L_{AC}=220\;\mathrm{mm}$. The base circle diameter D=100mm, and the cylindrical cam height H=50mm. The length of the pin is 14.48 mm, and the output stroke of the feed cylinder cam pendulum follower end is 14.5 mm, indicating that the stroke of the feed cam is 6.6 mm.

The stabilizing cam mechanism adopts a groove-type plane cam pendulum mechanism, as shown in Figure 5b. Combined with the cam timing relationship, pendulum $d_{1}=60\;\mathrm{mm}$, $d_{2}=100\;\mathrm{mm}$. The outer diameter of the plane cam is 100 mm and the base circle diameter is 60 mm. The cam base circle radius is 30 mm and the maximum stroke radius is 32.1 mm.

**Figure 6:**
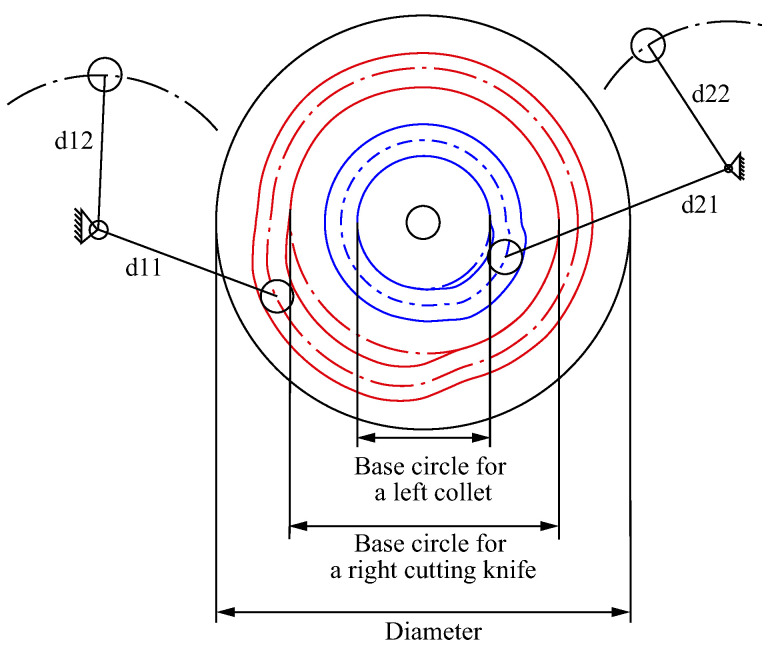
Clamping mechanism sketch.

The clamping and shearing module adopts a double-groove planar cam swing follower mechanism with a left collet groove and a right cutting groove. The structure sketch is shown in Figure 6. The left collet cam base circle radius is 35 mm, the maximum stroke radius is 43 mm, and the follower pendulum $d_{1}=80\;\mathrm{mm}$, $d_{2}=100\;\mathrm{mm}$. The base circle radius of the cam right cutting knife is 70 mm, and the maximum stroke radius is 76.4 mm. follower pendulum $d_{21}=110\;\mathrm{mm}$, $d_{22}=110\;\mathrm{mm}$.

The pin insertion module uses a cylindrical cam swing follower mechanism, as shown in Figure 5a. The roller on the cylindrical cam is located at point B of the pendulum, $L_{AB}=100\;\mathrm{mm}$, $L_{BC}=120\;\mathrm{mm}$. Then, the pendulum length $L_{AC}=220\;\mathrm{mm}$. The base circle diameter D=100mm. The stroke of the pendulum is 21 mm and the maximum stroke of the feed cam is 9.55 mm.

**Figure 7:**
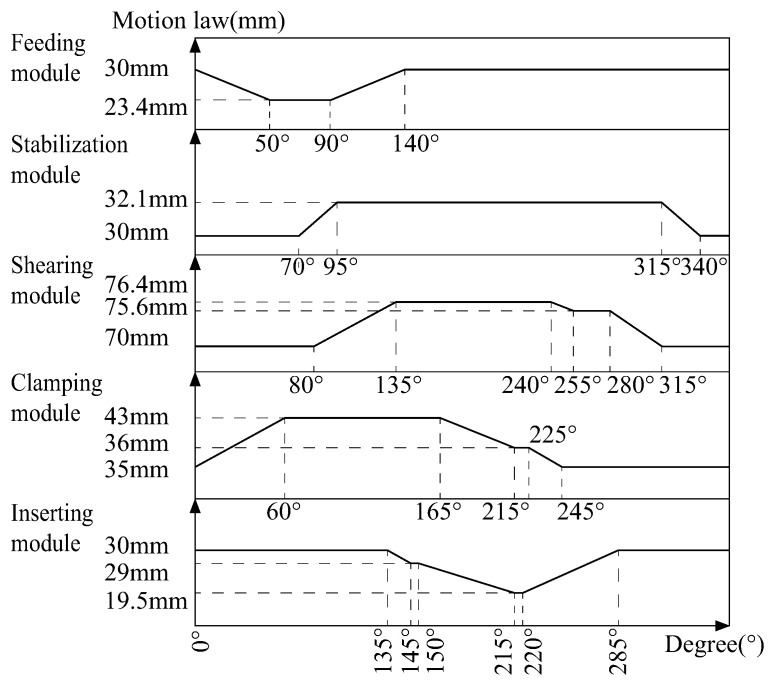
The motion laws of each cam in the cam insertion mechanism.

The four modules work in tandem to produce one pin in one rotation cycle. The length of a standard pin is 14.5 mm, and the designed productivity is 3 s/pin, that is, the spindle speed is 20 rpm. According to the motion requirements of the cam insertion mechanism, the final motion law is shown in Figure 7.

### 2.2. Cam Profile Curve Fitting and Solving

To ensure smooth and shock-free movement of the follower rollers in the cam pin mechanism, it is necessary to ensure that the cam profile, velocity, acceleration, and jerk curves are continuous and that the pressure angle is within a certain permissible range [32,33]. A seventh-degree polynomial can accommodate eight variables, which allows it to satisfy eight boundary conditions, including the position, velocity, acceleration, and jerk at both the starting and ending points. While higher-order polynomials can incorporate more variables, they also present greater challenges in terms of solving. Therefore, to strike a balance between jerk continuity and computational complexity, a seventh-degree polynomial motion profile is chosen for fitting to achieve an optimal motion state. The parameters involved in the cam curve solution are shown in Table 1.

The equation for the variation in the cam pendulum swing angle with respect to the transposition angle adopts a seventh-degree polynomial-type motion law curve. The general form of the swing angle equation is shown in Equation (1). To avoid redundancy, the feed cam solution is illustrated as an example.

(1) $$\theta =c_{0}+c_{1}\varphi +c_{2}\varphi^{2}+\ldots +c_{7}\varphi^{7}$$

According to Figure 7, the lift stage of the feeding cam is from 0 degrees to 50 degrees, and the numerical boundary conditions at its four points are shown in Equation (2).

(2) $$\left\{\begin{array}{rrrrr} \varphi_{f}=0; & \; & \theta_{f}^{\prime }=0; & \; & \theta_{f}^{\prime \prime }=0;\\ \varphi_{f}=20; & \; & \theta_{f}^{\prime }=-1.841; & \; & \theta_{f}^{\prime \prime }=-66.265;\\ \varphi_{f}=25; & \; & \theta_{f}^{\prime }=-2.081; & \; & \theta_{f}^{\prime \prime }=0;\\ \varphi_{f}=49; & \; & \theta_{f}^{\prime }=-0.0001; & \; & \theta_{f}^{\prime \prime }=2.121; \end{array}\right.$$

Substituting Equation (2) into Equation (1), the equation for the lift stage curve of the feeding cam is obtained as shown in Equation (3).

(3) $$\begin{array}{cc} \theta_{f}= & 0.1634\varphi_{f}^{3}+0.8794\varphi_{f}^{4}-0.0672\varphi_{f}^{5}\\ \; & -1.257\times 10^{-6}\varphi_{f}^{6}+0.0016\varphi_{f}^{7} \end{array}$$

According to Figure 7, the return stage of the feeding cam is between 90 degrees and 140 degrees. The return phase curve boundary is shown in Equation (4). The equation of the return phase curve of the feed can be solved as shown in Equation (5).

(4) $$\left\{\begin{array}{rrrrr} \varphi_{f}=90; & \; & \theta_{f}^{\prime }=0; & \; & \theta_{f}^{\prime \prime }=0;\\ \varphi_{f}=110; & \; & \theta_{f}^{\prime }=1.841; & \; & \theta_{f}^{\prime \prime }=66.265;\\ \varphi_{f}=115; & \; & \theta_{f}^{\prime }=2.081; & \; & \theta_{f}^{\prime \prime }=0;\\ \varphi_{f}=139; & \; & \theta_{f}^{\prime }=0.0001; & \; & \theta_{f}^{\prime \prime }=-2.121; \end{array}\right.$$

(5) $$\begin{array}{cc} \theta_{f}= & -3.196\times 10^{9}+1.3739\times 10^{8}\varphi_{f}-1.8846\times 10^{6}\varphi_{f}^{2}\\ \; & +0.0747\varphi_{f}^{3}+256.68\varphi_{f}^{4}-2.7517\varphi_{f}^{5}\\ \; & -2.042\times 10^{-6}\varphi_{f}^{6}+0.0122\varphi_{f}^{7} \end{array}$$

On the basis of the above process, the entire feed cam pendulum angular motion curve is derived, as shown in Table 2.

Similarly, the equations for the angular motion curves of the pendulum follower of the steady cam, the left collet of the clamping cam, the right cutting knife of the clamping cam, and the pin insertion cam curves can be obtained as shown in Table 3, Table 4, Table 5 and Table 6.

The parameter conditions of the polynomial law of motion curve-fitting motion are shown in Table 7, in which the pressure angle of the left collet of the clamping cam is the largest, with a pressure angle of 36.28°. The pushing pressure angle of the moving follower disc cam mechanism should not exceed 35°, and the swinging follower can be relaxed to 45°. During the return phase, it is allowed to reach 70° [34]. Therefore, 36.28° is smaller than the pressure angle [α] = 70° required for the cam pendulum mechanism.

**Figure 8:**
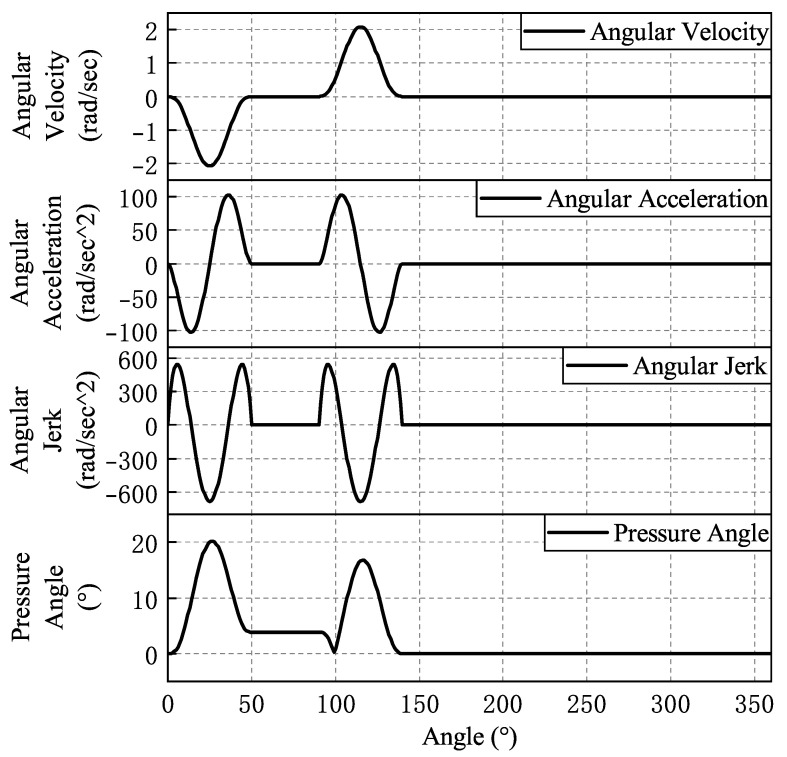
Feeding cam motion curve.

**Figure 9:**
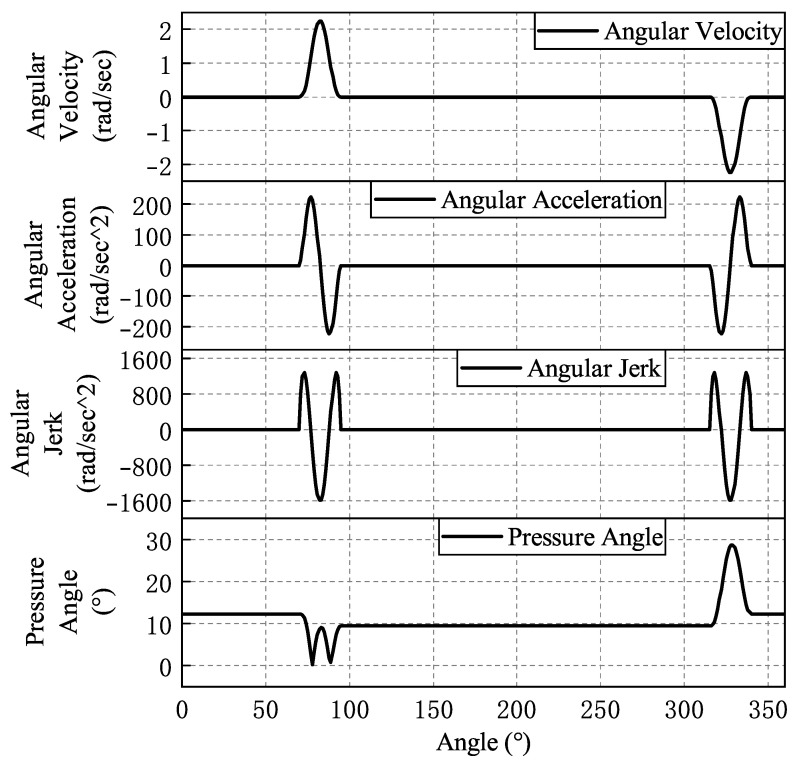
Stabilizing cam motion curve.

**Figure 10:**
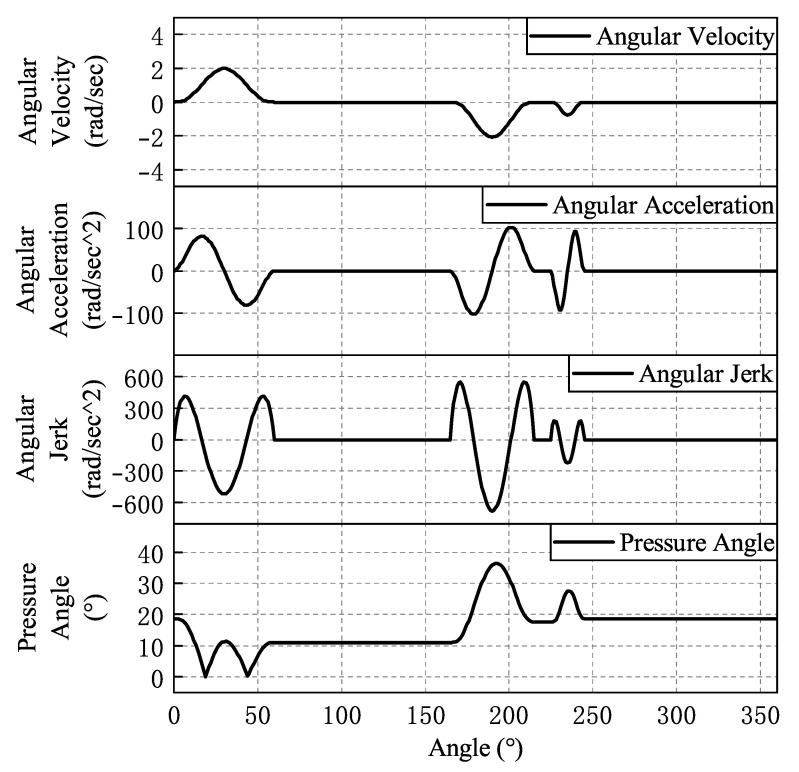
The left collet of the clamping cam motion curve.

**Figure 11:**
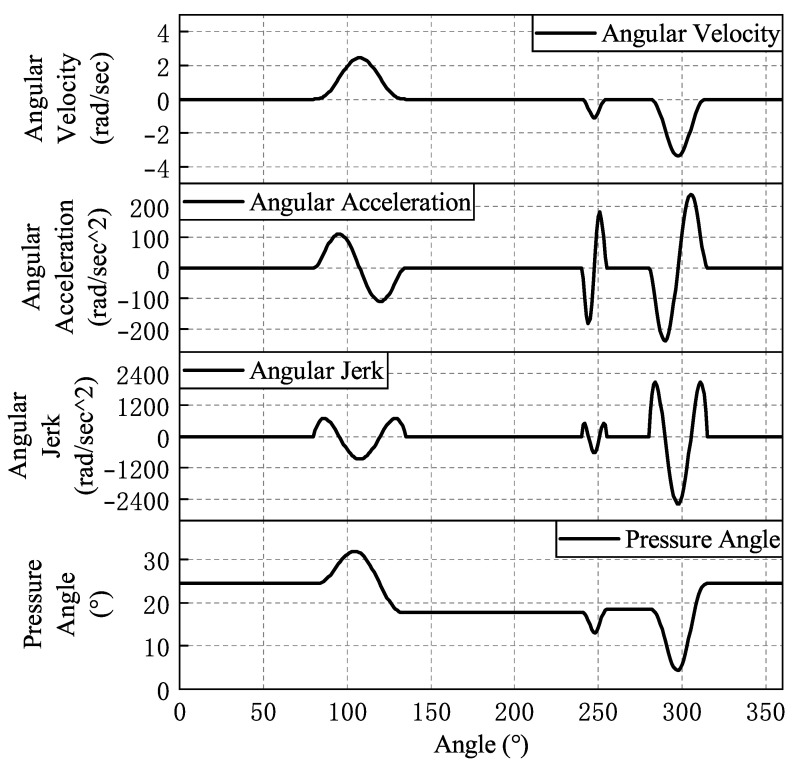
The right cutting knife of the clamping cam motion curve.

**Figure 12:**
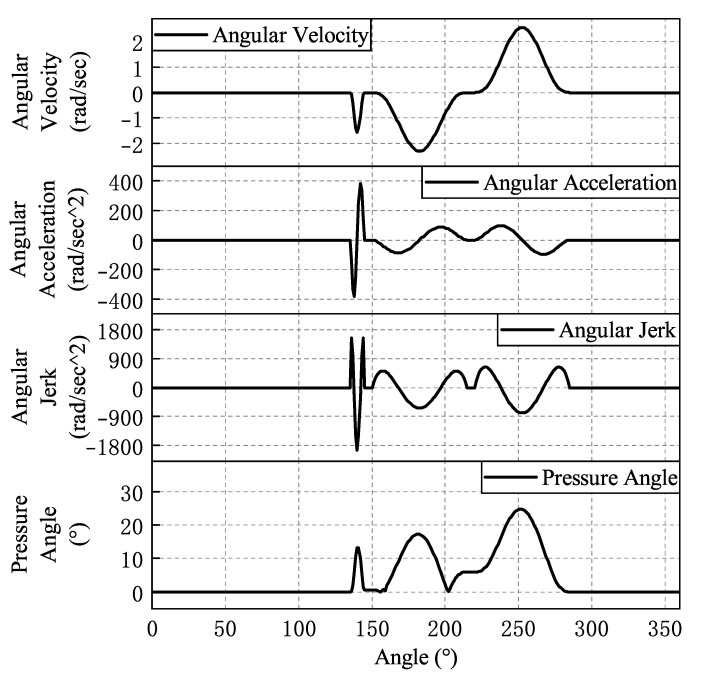
Pin inserting cam motion curve.

The fitted law of motion curves for each cam is shown from Figure 8, Figure 9, Figure 10, Figure 11 and Figure 12. In the whole cam insertion mechanism, the speed and acceleration curves of each cam are continuous, the pressure angle is within the allowable range, and the jerk is also within a certain range without sudden change.

## 3. Motion Simulation Verification of Cam Pin Mechanism

The purpose of motion simulation is to detect interference problems during the motion of the pin insertion cam group model and to ensure that the motion patterns of the individual cam mechanisms match the predefined timing. In addition, it aims to calculate the maximum angular velocity of the pin insertion module and verify the maximum insertion force of the cam pin insertion mechanism. Common modeling methods are graphical, parametric analysis, and computer-aided methods [34]. The computer-aided method combines the advantages of graphical and parametric analysis methods with computer simulation technology and uses computer-aided design software to implement the process.

### 3.1. Simulation Model Construction

**Figure 13:**
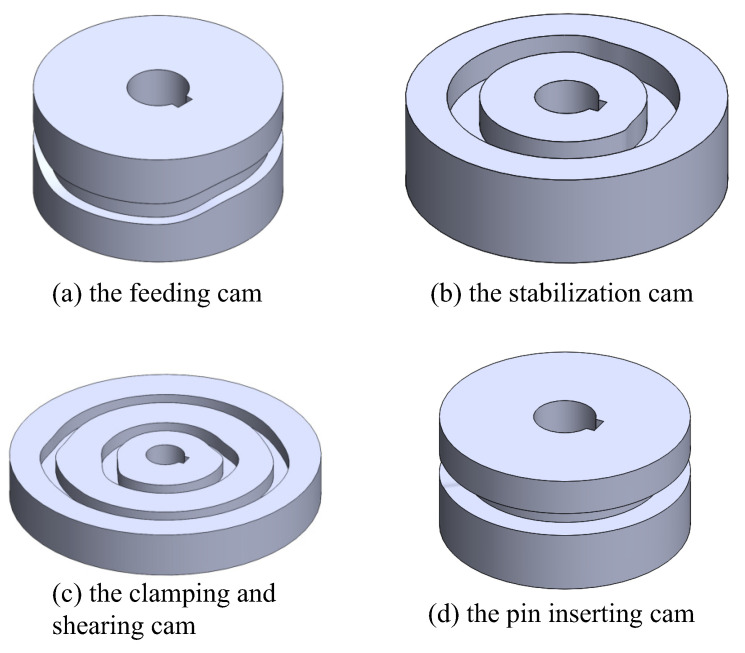
Polynomial curve-fitting cam models.

The cam models were modeled using SOLIDWORKS^®^ 3D CAD Software in conjunction with the CamTrax plug-in. The CamTrax plug-in automatically generates the required cylindrical cam models by simply inputting the basic cam parameters, such as the outer cylindrical surface diameter, roller diameter, roller width, cam groove depth, and lift data. All cam models are treated as rigid bodies. Figure 13 illustrates the models of the feeding cam, the stabilization cam, the clamping and shearing cam, and the pin inserting cam obtained using seventh-degree polynomial curve fitting.

**Figure 14:**
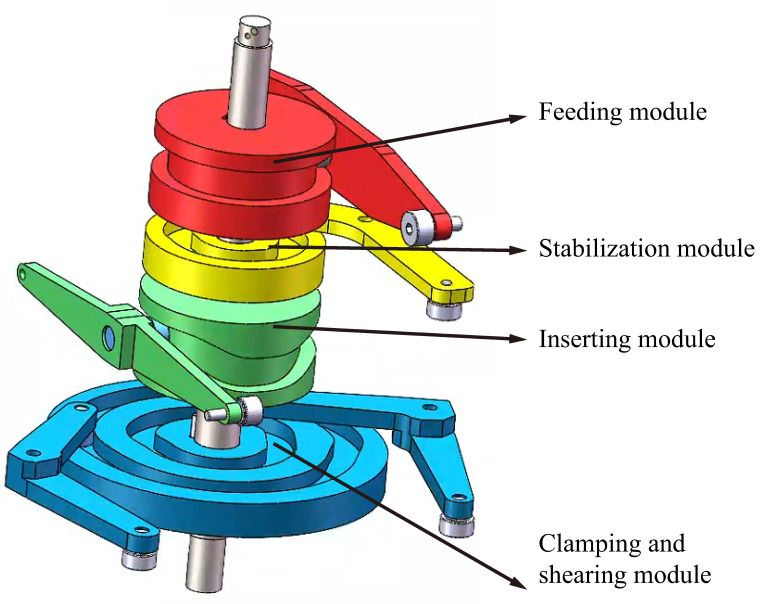
Simulation cam mechanism model.

A simplified model of the mechanism was created in SolidWorks for kinematic simulation, as shown in Figure 14. The ratio of the 3D model used for theoretical design to the actual object is 1:1. Components such as the housing are removed to reduce the computational load on the computer.

**Figure 15:**
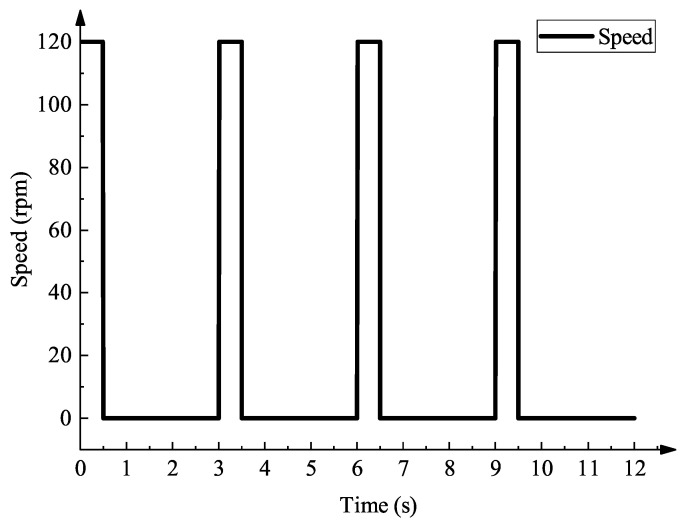
Motor simulation speed.

The SolidWorks Motion module is used to simulate the kinematics of the cam pin insertion mechanism by inputting the drive mode and the speed of the drive shaft. To conform to the use of the motor in the actual production process, the motor speed is set as a variable speed motion, using a motion cycle of 120 rpm with a hold of 0.5 s and a pause of 2.5 s. The total motion time is 12 s, and the speed curve is shown in Figure 15. The center of mass of the follower roller in the cam mechanism is used as the reference point for linear velocity and linear acceleration to compare the theoretical motion curves obtained by camtrax with the simulated motion curves obtained by the motion module.

### 3.2. Analysis of Simulation Results

Based on the motion data of each module obtained by simulation, the linear velocity and linear acceleration curves of the pendulum follower rollers of each cam are obtained. Since the fitting results in Section 2.2 are the angular velocities and angular accelerations of the pendulum follower rollers of each cam, it is necessary to convert them into units for comparison.

The angular velocity and velocity conversion relationship is shown in Equation (6).

(6) $$\begin{array}{c} v=r\omega \end{array}$$

where *r* is the radius of rotation, *v* is the linear velocity, and ω is the angular velocity.

The angular acceleration α and the linear acceleration *a* conversion relationship are shown in Equation (7).

(7) $$\begin{array}{c} a=r\alpha \end{array}$$

Each cam pendulum mechanism has a different pendulum length. According to Section 2.1, the linear velocity and linear acceleration at the end of each cam pendulum mechanism are known, and the angular velocity and angular acceleration are converted accordingly.

**Figure 16:**
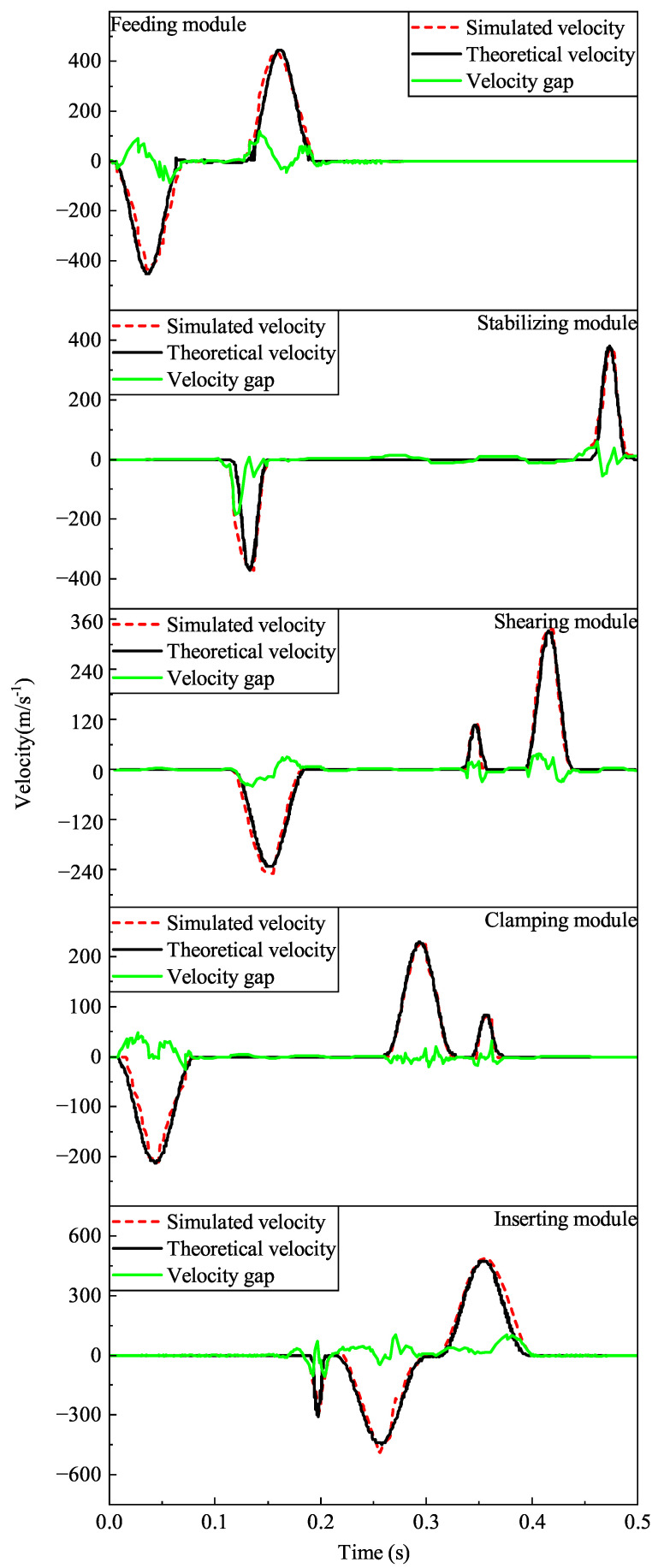
Comparison of simulated and theoretical velocity values for each module of the mechanism.

**Figure 17:**
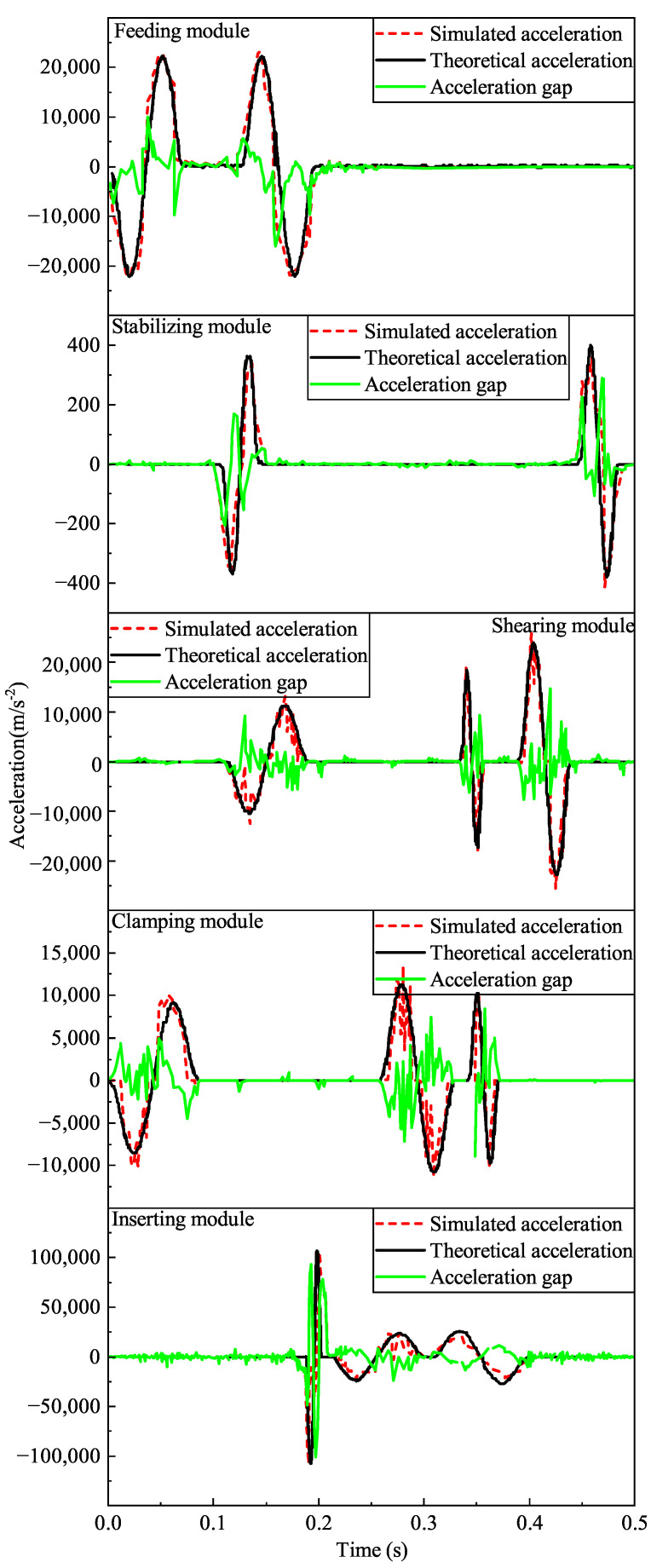
Comparison of simulated and theoretical acceleration values for each module of the mechanism.

Motion simulation comparison results show that the simulated motion curves conform to the fitted curves, as shown in Figure 16 and Figure 17. Due to the time difference between the lift and return, the gap is relatively large, but it is quickly smoothed out. Since there is a gap between the follower wheel and the cam groove, some minor jumps occur, but they are within reasonable limits. There are no large abrupt changes in velocity or acceleration, so the polynomially fitted curves meet the design requirements. The acceleration theoretical profile in the simulation of the pin inserting cam motion has the acceleration and leap change amplitude by the theoretical law of motion curve. The rationality of the cam pin insertion mechanism is further verified.

## 4. IGBT Pin Defect Detection and Pin Insertion Alignment

**Figure 18:**
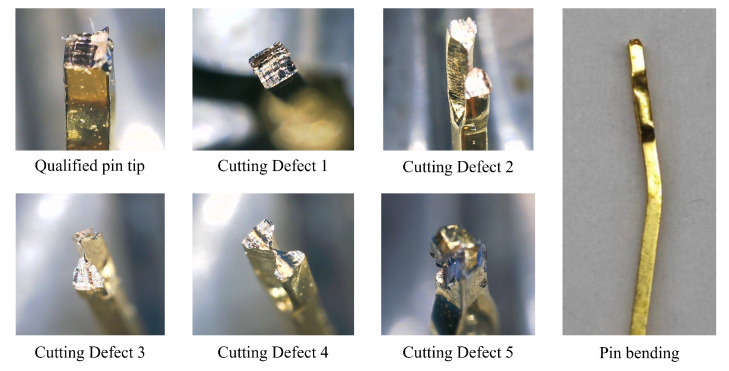
Qualified pins and their defects.

**Figure 19:**
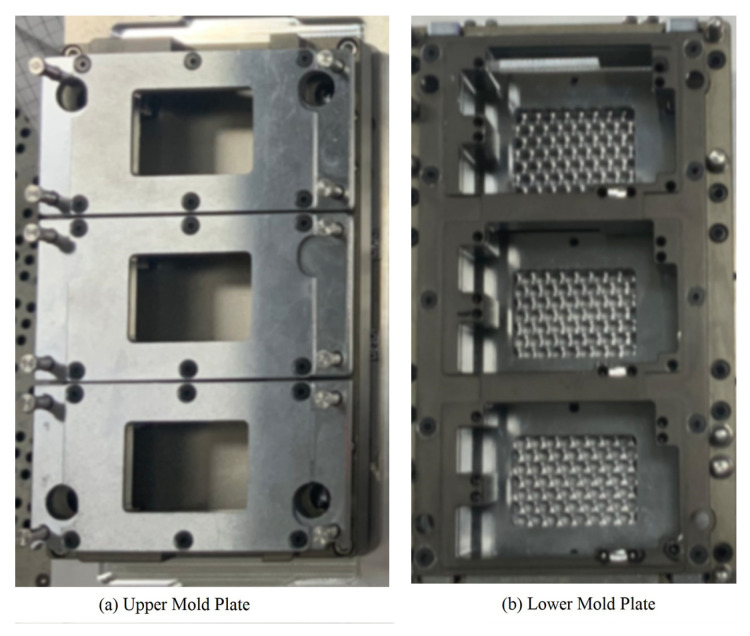
Pin mold.

The pins are cut by the pin insertion mechanism. During the shearing process, various defects may appear on pins. The comparison between the qualified pins and the defective pins is shown in Figure 18. The pin mold is used for guiding and fixing the pins before reflow soldering, as shown in Figure 19.

**Figure 20:**
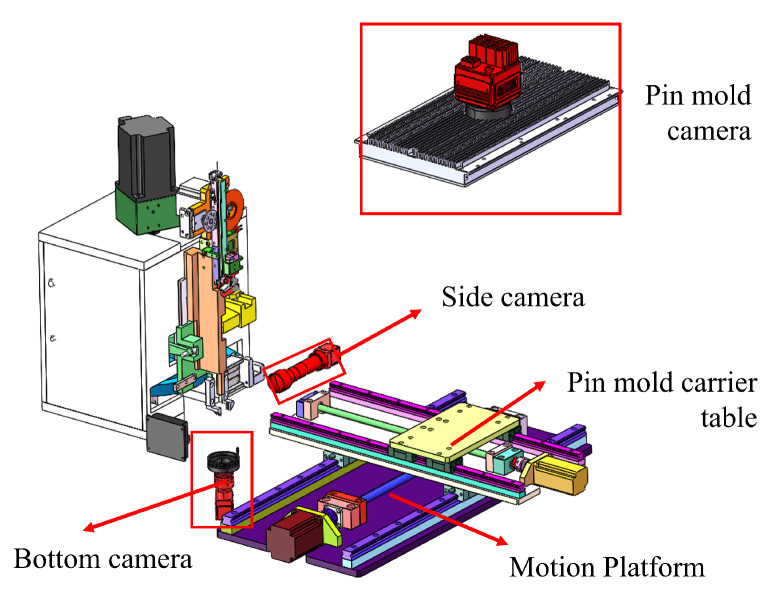
The schematic diagram of the visual system.

By collecting images of the pin tip and the side of the pin body, defects can be detected. Combined with the image of the pin mold, visual guidance of the pin position is achieved. The schematic diagram of the visual system is shown in Figure 20.

**Figure 21:**
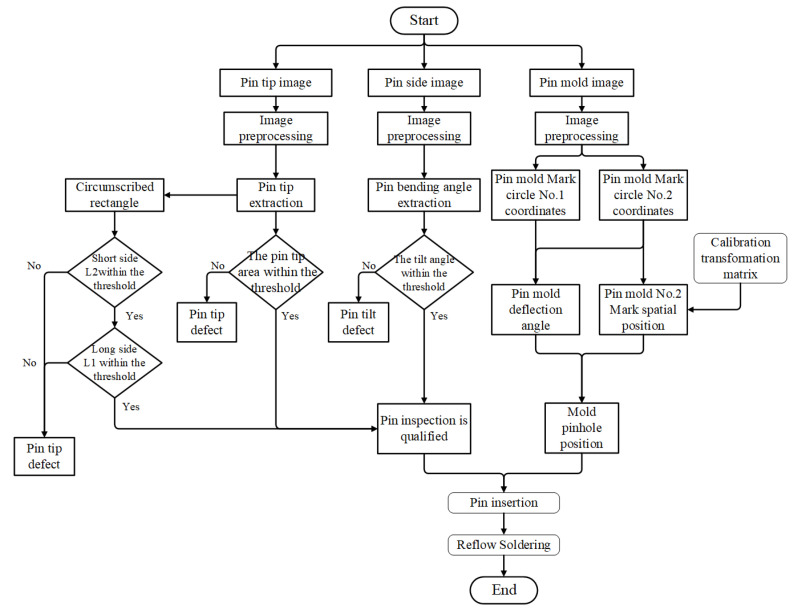
Framework of pin defect inspection and insertion.

The visual algorithm framework for detecting pin defects and aligning pin insertion is illustrated in Figure 21. Pin tip defects, pin bending, and pin insertion alignment are discussed in three sections.

### 4.1. Pin Tip Defect Detection

**Figure 22:**
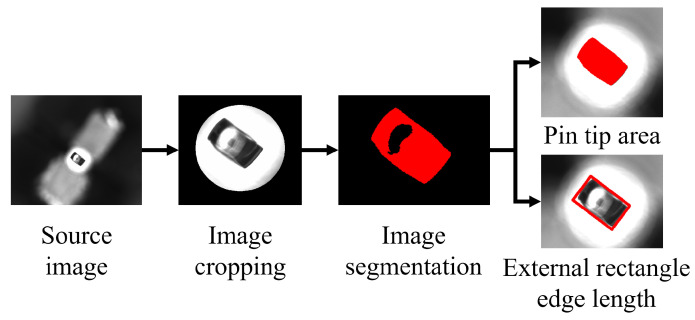
Pin bottom image and preprocessing process.

The image of the pin tip captured by the camera contains a large amount of noise, which must be cropped, segmented, and processed within the pin tip domain before the defect judgment result can be calculated, as shown in Figure 22. The information required for determining pin tip defects includes two points: the top area of the tip and the external rectangular parameters of the tip.

The visual algorithm is established using the HALCON platform. It contains multiple visual operators, with strong compatibility and flexibility. The HOperatorSet.ReduceDomain operator is used to complete image cropping. The detection target for IGBT pin detection is singular, and the environment is stable. Therefore, the HOperatorSet. Threshold operator is employed to perform image segmentation through threshold binarization. This method is straightforward to implement, has low computational complexity, and is highly efficient; however, it is sensitive to noise and exhibits low robustness. The segmented pin tip area is filled and finally the HOperatorSet is used. The ReaCenter operator is employed to determine whether area S of this section is within the acceptable range. The HOperatorSet.SmallestRectangle2 operator is called to obtain lengths L1 and L2 of the two sides of the bounding rectangle, and its parameters are used to determine whether the long side L1 and the short side L2 are within the specified threshold.

### 4.2. Pin Tilt Detection

**Figure 23:**
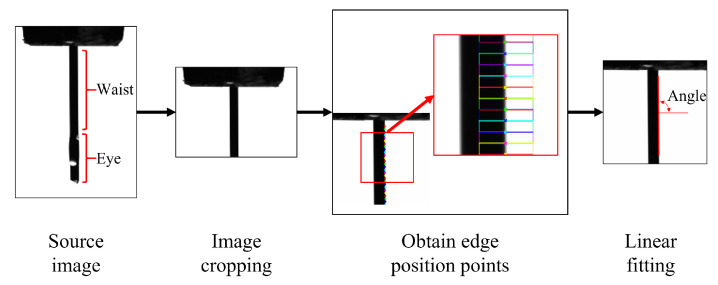
Pin tilt detection.

The tilt angle of the pin is extracted from the side image of the pin to determine whether the pin is tilted or bent. When the angle between the pin and the horizontal line is not between 88° and 92°, it is determined that the pin is tilted. The core algorithm for inclination detection is to calculate the inclination of edge lines through edge detection algorithms and line fitting algorithms. The algorithm processing flow is shown in Figure 23.

First, the waist image of the pin is cropped to ensure visual detection accuracy and reduce computation. Then, the Gaussian filtering function is used to determine edge detection points [35]. Finally, for each fixed edge detection point, a straight line fitting is performed to obtain the angle between the fitted line and the horizontal line. The tilt angle is then assessed for qualification.

### 4.3. Pin Insertion Alignment

**Figure 24:**
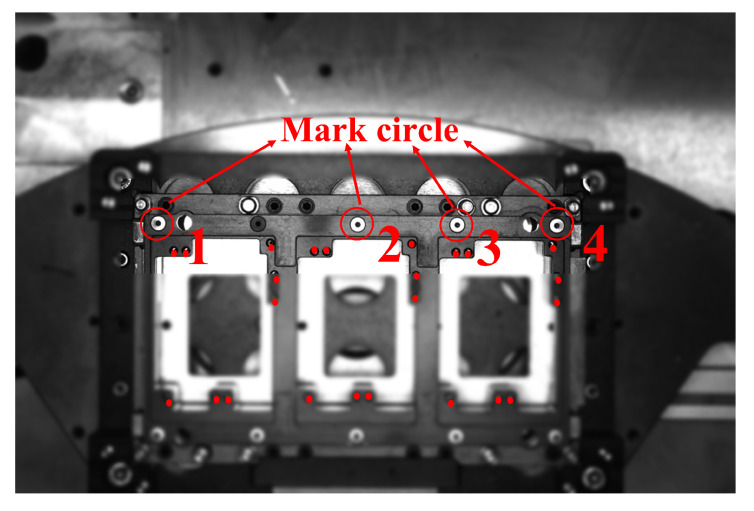
Mark circle on the pin mold.

After the pin defect detection is qualified, the pin can be guided and positioned into the pinhole. The edge fitting positioning scheme is used to obtain the center position of mark circle on the pin mold, as shown in Figure 24. Then, based on the relative position relationship between the mark circle and the pinhole, the actual coordinates of the pinhole are obtained. The motion platform moves accordingly according to this coordinate information and finally completes the pin guidance work.

**Figure 25:**
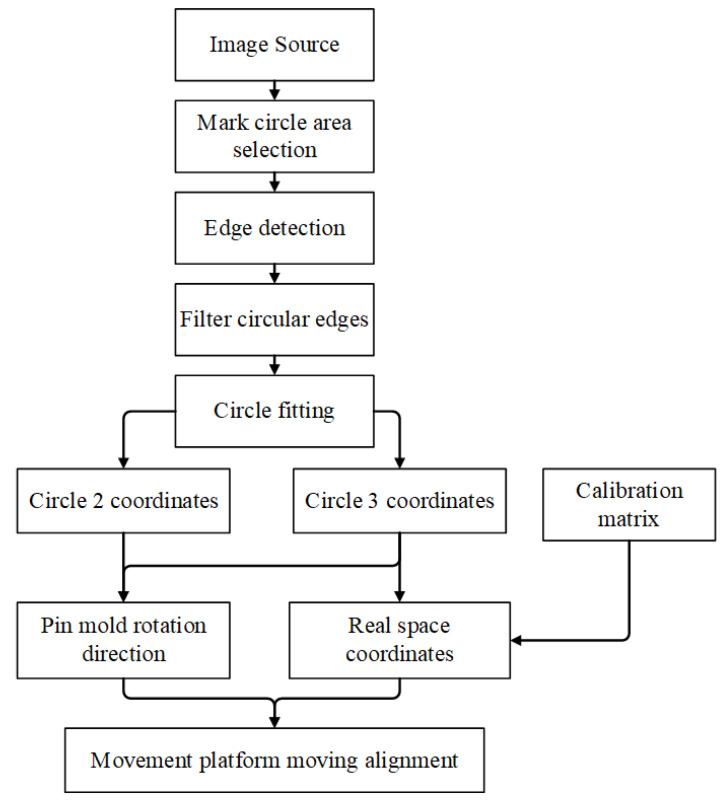
Extraction process of center coordinates of pin mold mark circle.

The specific steps for pin insertion alignment are illustrated in Figure 25. First, the excess parts are trimmed off to improve computational efficiency, and then two mark circles are cut out in the image. Then, the threshold binary image segmentation method is used to select the image domain of the small area surrounding the marked circles, and the binary mark circle discrete connected domain is filtered. Then, Canny combined with Gaussian filtering is used for edge detection of marked circles. Gaussian filtering effectively removes small noise and the Canny edge detection operator has better noise suppression capabilities compared to the Laplace and Sobel operators. Additionally, the Tukey geometric fitting algorithm is employed to determine the center of the mark circle. The angle between two points, calculated using the center coordinates of Mark Circle 2 and Mark Circle 3, represents the deflection angle of the pin mold. By substituting the camera calibration matrix, the actual spatial position coordinates of Mark Circle 2 can be obtained.

### 4.4. Pin Height Detection

Measuring the height of the pin can further verify the timing stability and pin yield of the cam pin insertion mechanism. Due to the size of IGBT modules, traditional 2D cameras are prone to distortion. Structured light vision-based line laser sensors are used to measure pin height in sections and batches.

**Figure 26:**
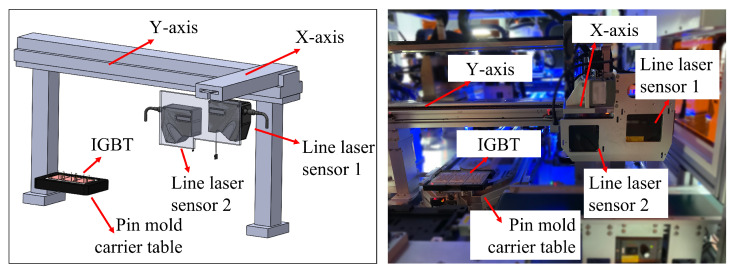
IGBT module pin height measurement platform.

Due to the varying degrees of warping of the same reference surface after the reflow soldering process and the different positions of the pins in the IGBT module, the height of the warping is also different. Therefore, each pin uses its own reference surface to calculate its height. Two laser sensors with staggered height layout are used to collect the depth point cloud at the bottom of the IGBT module and the point cloud at the tip of the pin. The designed detection platform is shown in Figure 26. The line laser sensor mechanism is installed on a moving platform with X-axis and Y-axis movement directions, which enables the line laser sensor mechanism to complete point cloud acquisition work according to segmented acquisition paths. The X and Y moving axes complete the line scanning work according to the specified route under the drive of the motor. The measurement program for pin height is based on HALCON, and the specific algorithm can be referred to in our previous work [36].

## 5. Onboard Testing

**Figure 27:**
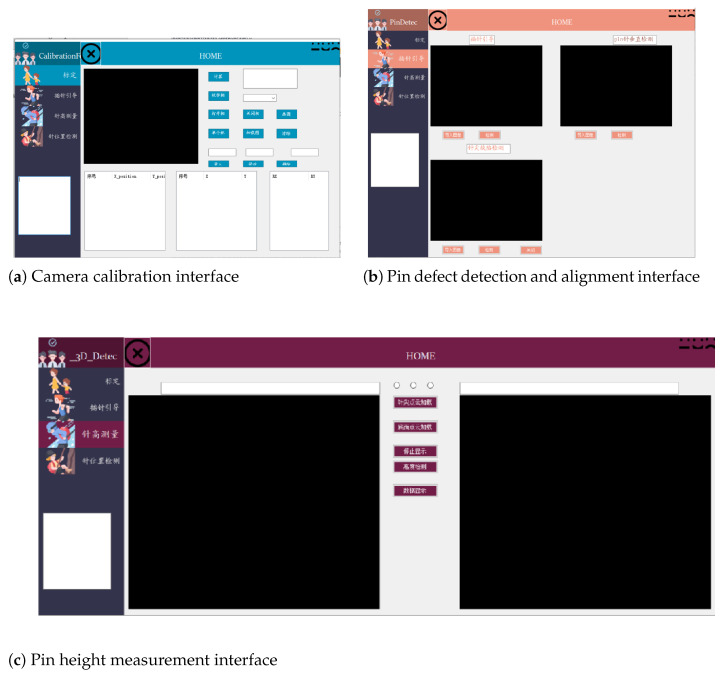
Human–computer interaction platform.

To facilitate the verification of the performance of each part of the system, all visual algorithms are embedded into a set of human–computer interaction platforms, as shown in Figure 27. The system includes camera calibration system, pin vision system, and pin height measurement system. The interface of the pin visual system includes three parts: pin tip defect detection, pin side defect detection, and pin mold visual guidance.

The cam insertion mechanism, defect detection algorithm, and visual guidance algorithm are integrated to create an insertion machine. Considering the specific camera model and the pixel accuracy, the actual image recognition guidance accuracy achieves 0.02 mm, which is higher than the theoretical accuracy of 0.05 mm in the literature [15]. The positional accuracy between the mark circle and the pinholes of the pin mold is 2 µm, and the range of repeated measurements for the pin height is less than or equal to 0.04 mm. The working environment of the packaging equipment is stable, so the actual achievable accuracy is close to 0.02 mm, which is better than the accuracy of 0.06 mm or 0.09 mm in the literature [14,17].

To evaluate the efficiency, accuracy, stability, and reliability of the packaging device, experimental tests were conducted to measure the pin insertion time, pin output yield, and the qualification rate of the produced products from the pin insertion machine.

(1) Pin yield detection

**Figure 28:**
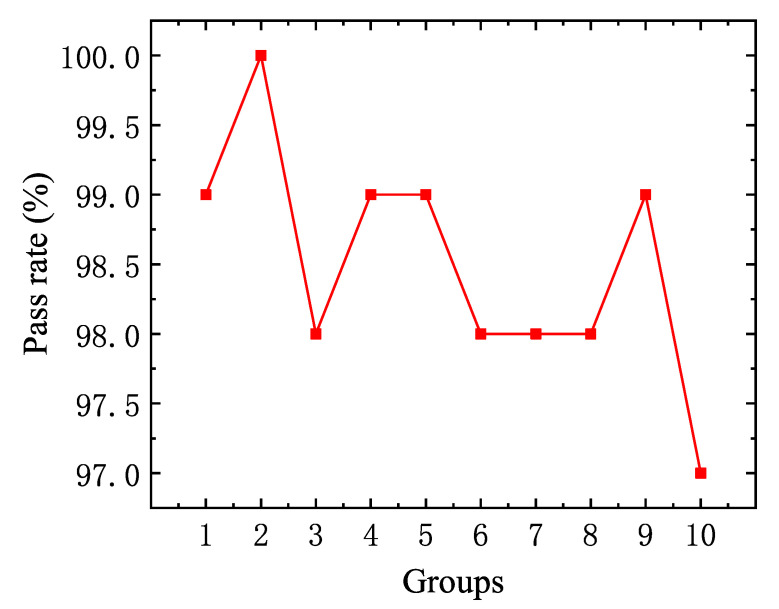
Ten sets of pin incoming inspection pass rates.

The visual algorithm program can be used to statistically analyze the NG/OK signals from incoming inspection of pins to determine the yield of pin cutting in the cam pin mechanism. In this experiment, 1000 visually inspected pins were cut using the pin mechanism to assess whether they were classified as NG (No Good) or OK (Good). The 1000 pins were evenly divided into ten sets of experimental data, and the pin tip inspection pass rate is illustrated in Figure 28, which shows a pass rate exceeding 97%.

(2) Pin insertion time verification

**Figure 29:**
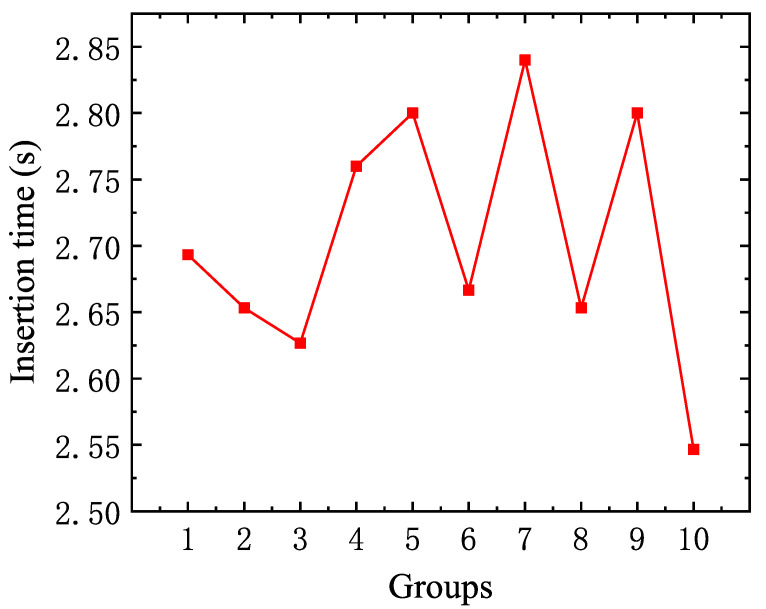
Ten sets of pin insertion times.

The insertion rate index reflects the efficiency, stability, and reliability of the visual algorithm used in the insertion mechanism. To evaluate this, 1,000 pins were continuously produced using the cam pin insertion mechanism, organized into groups of 100 pins each. The data were divided into ten groups, and the average pin insertion time for each group was calculated. As illustrated in Figure 29, the maximum insertion time recorded for the insertion mechanism, when combined with the visual algorithm platform, was 2.84 s per pin, same as the detection speed of less than 3 s in the literature [37].

(3) Pin qualification rate experiment

**Figure 30:**
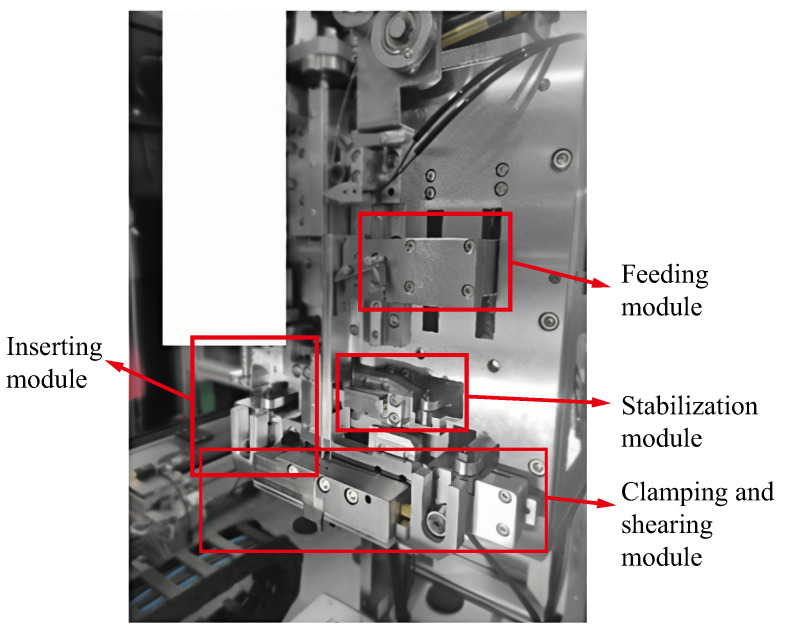
Cam pin insertion mechanism.

To further verify the stability of the cam pin insertion mechanism as well as the reliability of the timing, after detecting pin tip defects, bending defects, and height defects, the qualification rate of IGBT module pins produced by the cam pin mechanism is assessed by measuring the high qualification rate of the product pins. The cam pin insertion mechanism is shown in Figure 30. The standard length of a single pin is 14.5 mm, and considering the thickness of the solder paste, the standard height range of the pin on the IGBT module is 15.5 ± 0.4 mm.

**Figure 31:**
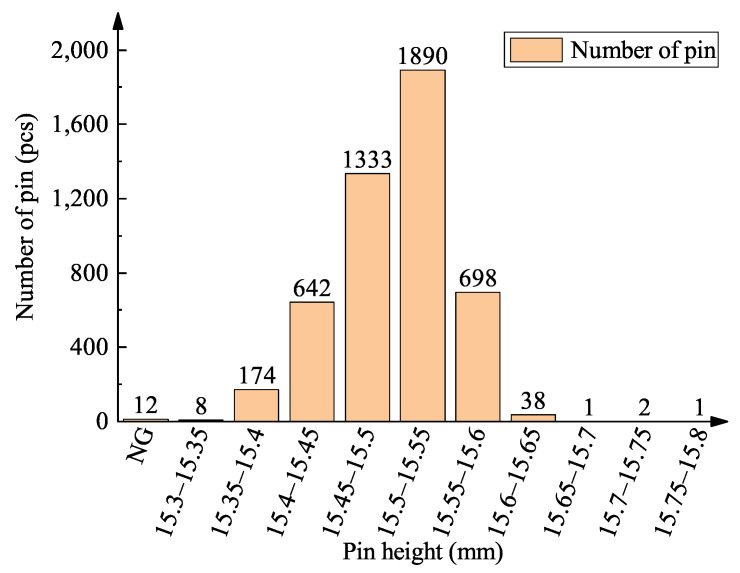
Pin height distribution.

**Figure 32:**
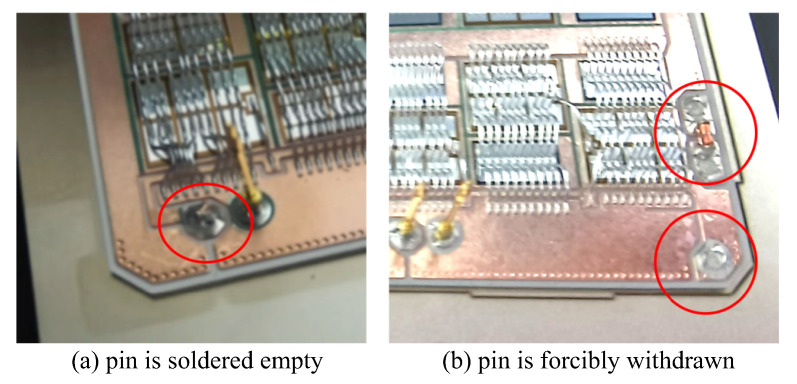
Pin missing defects.

The cam pin insertion mechanism is assembled on the machine to produce 200 IGBT modules, and each IGBT module has 24 pins, totaling 4800 pins. With the help of the pin height measurement platform, the pin height distribution of the IGBT module is measured, as shown in Figure 31. Twelve pins lost height data, and the heights of the remaining pins were within the range of 15.5 ± 0.4 mm. The main interfering factors that cause the loss of pins are as follows:

(1) The pin mold cannot fix the pin effectively or the pin is not fixed well, resulting in the pin being soldered in the IGBT module in the flip transfer process falling, and the solder paste is full of empty solder state, as shown in Figure 32a.

(2) The cam pin insertion machine cannot successfully insert the pin into the pin mold, resulting in solder paste on the DBC but no pin. However, there is a pin assembly completion test in the pin assembly line. When the number of pins inserted into the pin mold is not 24, the NG signal is reported, the production is paused, and the pin assemble is handed over to the manual secondary inspection.

(3) Poor pin assembly due to design defects of the pin mold resulted in a pin jamming problem in the pin mold after soldering. The pin was forcibly pulled out from the DBC during mechanical disassembly of the pin mold, resulting in the pin missing. The defective pattern is shown in Figure 32b.

A total of 12 defective pins were inspected and analyzed, and the results are shown in Table 8. Eight pins missing soldering occurred on the same pin mold, and the solder paste on this pin mold was full without soldering, as shown in Figure 32a. This is because after the pin insertion is completed, the reflow soldering process needs to be used to solidify the solder at the bottom of the pin, and the solder of the IGBT was not completely melted, resulting in the failure of pin fixation. Therefore, this is a reflow soldering defect. Additionally, four IGBT modules with a total of four pins became stuck during the pin mold disassemble process after the reflow soldering process, resulting in pin failure when pulled out the mold. The solder paste status is shown in Figure 32b. The leakage detection of the height measurement platform is zero. These 12 pins are all considered NG pins in this paper to account for the experimental test extremes. The height yield of the pin reaches 99.75% ((4800−12)/4800=99.75%), which is higher than 95.4% in the literature [38].

Considering the effects of pin assembly, DBC, solder pads, pin molds, etc., the high yield of the cam pin insertion mechanism for pins is 99.75% because of the inability to retrieve the eight dropped pins and the four forcibly pulled out pins. This yield rate directly verifies the timing stability and reliability of the cam pin insertion mechanism. This further verifies the reasonableness of the cam’s contour-fitting curve design.

## 6. Conclusions

This article presents the design of contour curves for four cams used in an IGBT pin insertion machine, utilizing a seventh-order polynomial and time series decomposition. Through simulation and physical verification, the feasibility and stability of employing the seventh-order polynomial for designing the contours of cylindrical and planar cams is proven. By developing image recognition algorithms, we successfully implement detection for pin tilt defects, pin tip defects, and pin insertion guidance. Additionally, we construct and test a pin insertion machine along with its human–machine interaction interface on actual machines. The experimental results indicate that the qualification rate for pin cutting reaches 97%. The average insertion time for each pin is 2.84 s, which aligns with existing literature. The qualification rate for pin insertion achieves 99.75%. Furthermore, the image guidance accuracy for the pin insertion position reaches 0.02 mm, suggesting that the design of the cam pin insertion mechanism is sound. In the future, the cam design curve can be refined through optimization to further enhance the stability and performance of the system.

## Figures and Tables

**Table 1:** Initial setting parameters.

Cams	Angular Displacement of Oscillating Parts	Cam Angle	Polynomial Coefficients
Feed cams	$\theta_{f}$	$\varphi_{f}$	$c_{fi}$
Stabilising cams	$\theta_{s}$	$\varphi_{s}$	$c_{si}$
Clamping cam left cutter	$\theta_{cl}$	$\varphi_{cl}$	$c_{cli}$
Clamping cam right cutter	$\theta_{cr}$	$\varphi_{cr}$	$c_{cri}$
Pin insertion cams	$\theta_{p}$	$\varphi_{p}$	$c_{pi}$

**Table 2:** Seventh-degree polynomial fitting curves of the feeding cam.

Stroke Phase	Seventh-Degree Polynomial Curve Equation	Turning Angle (°)
Lift Stage	$\theta_{f}=0.1634\varphi^{3}+0.8794\varphi^{4}-0.0672\varphi^{5}-1.257\times 10^{-6}\varphi^{6}+0.0016\varphi^{7}$	[0,50]
Far Rest Stage	$\theta_{f}=3.787$	[50,90]
Return Stage	$\theta_{f}=-3.196\times 10^{9}+1.3739\times 10^{8}\varphi -1.8846\times 10^{6}\varphi^{2}+0.0747\varphi^{3}+256.68\varphi^{4}-2.7517\varphi^{5}-2.042\times 10^{-6}\varphi^{6}+0.0122\varphi^{7}$	[90,140]
Near Rest Phase	$\theta_{f}=0$	[140,360]

**Table 3:** Seventh-degree polynomial fitting curves of the stabilizing cam.

Stroke Phase	Seventh-Degree Polynomial Curve Equation	Turning Angle (°)
Near Rest Phase	$\theta_{s}=0$	[0,70] U [340,360]
Lift Stage	$\theta_{s}=-9.1113\times 10^{5}+40124\varphi -527.03\varphi^{2}+6.9883\times 10^{-4}\varphi^{3}+0.0386\varphi^{4}-1.2797\varphi^{5}+1.1932\times 10^{-8}\varphi^{6}-2.8724\times 10^{-6}\varphi^{7}$	[70,95]
Far Rest Stage	$\theta_{s}=2.04$	[95,315]
Return Stage	$\theta_{s}=-3.3914\times 10^{9}+3.8664\times 10^{7}\varphi -132770\varphi^{2}+5.0207\times 10^{-5}\varphi^{3}+0.6863\varphi^{4}+3.3323\times 10^{-9}\varphi^{5}+4.436\times 10^{-9}\varphi^{6}-3.824\times 10^{-6}\varphi^{7}$	[315,340]

**Table 4:** Seventh-degree polynomial fitting curves of the left clevis of the clamping cam.

Stroke Phase	Seventh-Degree Polynomial Curve Equation	Turning Angle (°)
Lift Stage	$\theta_{cl}=0.11876\varphi^{3}+2.4984\times 10^{-3}\varphi^{4}-1.4826\times 10^{-3}\varphi^{5}-5.3196\times 10^{-7}\varphi^{6}+5.4282\times 10^{-6}\varphi^{7}$	[0,60]
Far Rest Stage 1	$\theta_{cl}=4.32$	[60,165]
Return Stage 1	$\theta_{cl}=5.1815\times 10^{7}-1.0327\times 10^{6}\varphi +6175.6\varphi^{2}+4.0648\times 10^{-4}\varphi^{3}-0.0955\varphi^{4}+4.7606\times 10^{-8}\varphi^{5}+33.0775\times 10^{-9}\varphi^{6}+1.5621\times 10^{-6}\varphi^{7}$	[165,215]
Far Rest Stage 2	$\theta_{cl}=0.55$	[215,225]
Return Stage 2	$\theta_{cl}=-2.5232\times 10^{9}-4.0127\times 10^{7}\varphi -1.922\times 10^{5}\varphi^{2}+2.0804\times 10^{-4}\varphi^{3}+1.9322\varphi^{4}-9.8456\times 10^{-9}\varphi^{5}+3.3846\times 10^{-8}\varphi^{6}-2.0929\times 10^{-5}\varphi^{7}$	[225,245]
Near Rest Phase	$\theta_{cl}=0$	[245,360]

**Table 5:** Seventh-degree polynomial fitting curves of the right cutter of the clamping cam.

Stroke Phase	Seventh-Degree Polynomial Curve Equation	Turning Angle (°)
Near Rest Phase	$\theta_{cr}=0$	[0,80] U [315,360]
Lift Stage	$\theta_{cr}=-3.2739\varphi^{6}+1.2627\times 10^{5}\varphi -1.45\times 10^{3}\varphi^{2}+1.3071\times 10^{-3}\varphi^{3}+0.0805\varphi^{4}+6.9949\times 10^{-7}\varphi^{5}+1.5916\times 10^{-8}\varphi^{6}-4.5027\times 10^{-6}\varphi^{7}$	[80,135]
Far Rest Stage 1	$\theta_{cr}=4.9$	[135,240]
Return Stage 1	$\theta_{cr}=1.0468\times 10^{1}0-1.5752\times 10^{8}\varphi +7.1411\times 10^{5}\varphi^{2}-3.6\times 10^{-4}\varphi^{3}-6.4363\varphi^{4}+1.263\times 10^{-8}\varphi^{5}-9.5904\times 10^{-8}\varphi^{6}-6.2572\times 10^{-5}\varphi^{7}$	[240,255]
Far Rest Stage 2	$\theta_{cr}=4.3$	[255,280]
Return Stage 2	$\theta_{cr}=3.0206\times 10^{9}-3.786\times 10^{7}\varphi +1.4285\times 10^{5}\varphi^{2}-3.4186\times 10^{-4}\varphi^{3}-0.8897\varphi^{4}-4.0352\times 10^{-9}\varphi^{5}-7.567\times 10^{-9}\varphi^{6}+5.9671\times 10^{-6}\varphi^{7}$	[280,315]

**Table 6:** Seventh-degree polynomial fitting curves of the inserting cam.

Stroke Phase	Seventh-Degree Polynomial Curve Equation	Turning Angle (°)
Far Rest Stage	θ=0	[0,135] U [285,360]
Lift Stage 1	$\theta =4055.5-102.64\varphi +0.408\varphi^{2}+4.1755\times 10^{-6}\varphi^{3}+1.2785\times 10^{-4}\varphi^{4}-1.8578\times 10^{-6}\varphi^{5}-1.658\times 10^{-11}\varphi^{6}+9.3738\times 10^{-9}\varphi^{7}$	[135,145]
Near Rest Phase 1	θ=−0.859	[145,150]
Lift Stage 2	$\theta =2.1962\times 10^{5}-4271.9\varphi +24.021\varphi^{2}-1.3576\times 10^{-4}\varphi^{3}-2.5739\times 10-4\varphi^{4}-1.8914\times 10^{-9}\varphi^{5}+6.8041\times 10^{-12}\varphi^{6}+1.1257\times 10^{-11}\varphi^{7}$	[150,215]
Near Rest Phase 2	θ=−6.027	[215,220]
Return Stage	$\theta =-1.0012\times 10^{7}+1.287\times 10^{5}\varphi -476.6\varphi^{2}+2.2157\times 10^{-4}\varphi^{3}-2.211\times 10^{-3}\varphi^{4}-9.691\times 10^{-9}\varphi^{5}-1.6568\times 10^{-11}\varphi^{6}+2.7032\times 10^{-11}\varphi^{7}$	[220,285]

**Table 7:** Fitting parameters of each cam motion curve.

Cams	Maximum Angular Velocity (rad/s)	Maximum Angular Acceleration (${\mathrm{rad}/s}^{2}$)	Maximum Angular Jerk (${\mathrm{rad}/s}^{3}$)	Maximum Pressure Angle (°)
Feed cams	2.1	102.9	683.9	20
Stabilizing cams	2.2	222.5	1583.8	28.6
Clamping cam left clevis	2.1	102.5	679.2	36.3
Clamping cam right cutter	3.4	237.9	2556.7	31.9
Pin insertion cams	2.5	384.1	1959.6	24.7

**Table 8:** Manual secondary inspection results.

Reason for Pin Missing	Number of Pins (pcs)	Number of Pin Molds
Pin empty welded	8	1
Pin was pulled out	4	4
Height measuring platform leakage	0	0

## Data Availability

Data are contained within the article.
